# Non-hierarchical Influence of Visual Form, Touch, and Position Cues on Embodiment, Agency, and Presence in Virtual Reality

**DOI:** 10.3389/fpsyg.2016.01649

**Published:** 2016-10-25

**Authors:** Stephen C. Pritchard, Regine Zopf, Vince Polito, David M. Kaplan, Mark A. Williams

**Affiliations:** ^1^ARC Centre of Excellence in Cognition and its Disorders and Department of Cognitive Science, Macquarie UniversitySydney, NSW, Australia; ^2^Perception in Action Research Centre, Faculty of Human Sciences, Macquarie UniversitySydney, NSW, Australia

**Keywords:** virtual reality, embodiment, agency, presence, virtual hand illusion, self-representation

## Abstract

The concept of self-representation is commonly decomposed into three component constructs (sense of embodiment, sense of agency, and sense of presence), and each is typically investigated separately across different experimental contexts. For example, embodiment has been explored in bodily illusions; agency has been investigated in hypnosis research; and presence has been primarily studied in the context of Virtual Reality (VR) technology. Given that each component involves the integration of multiple cues within and across sensory modalities, they may rely on similar underlying mechanisms. However, the degree to which this may be true remains unclear when they are independently studied. As a first step toward addressing this issue, we manipulated a range of cues relevant to these components of self-representation within a single experimental context. Using consumer-grade Oculus Rift VR technology, and a new implementation of the Virtual Hand Illusion, we systematically manipulated *visual form plausibility, visual–tactile synchrony*, and *visual–proprioceptive spatial offset* to explore their influence on self-representation. Our results show that these cues differentially influence embodiment, agency, and presence. We provide evidence that each type of cue can independently and non-hierarchically influence self-representation yet none of these cues strictly constrains or gates the influence of the others. We discuss theoretical implications for understanding self-representation as well as practical implications for VR experiment design, including the suitability of consumer-based VR technology in research settings.

## Introduction

Researchers have long puzzled over how best to describe and study the way we experience and represent ourselves. To gain traction on this problem, a common strategy is to decompose the concept of self-representation into several distinct components. These include: sense of embodiment—the experience of owning a body and knowing its location (Longo et al., 2008b); sense of agency—the experience of causing actions and events in the world (Wegner, 2004); and sense of presence—the experience of “being there,” of being situated in an environment (Sanchez-Vives and Slater, 2005).

These components of self-representation have typically been studied independently in a variety of different experimental contexts. For example, embodiment has been investigated using bodily illusions (Botvinick and Cohen, 1998; Longo et al., 2008b; Tsakiris, 2010; Blanke, 2012; Ehrsson, 2012; Blanke et al., 2015); agency has been manipulated using hypnosis techniques (Kihlstrom, 2008; Polito et al., 2014) and in clinical research (Frith and Done, 1989); and presence has been investigated in the context of Virtual Reality (VR) and communication technologies such as videoconferencing (e.g., Ijsselsteijn et al., 2001; Sanchez-Vives and Slater, 2005).

### Embodiment cues

One way to understand these components of self-representation (embodiment, agency, presence) is to explore how they are induced and modified by different sensory cues. For example, research into embodiment has largely focused on the rubber hand illusion (RHI, Botvinick and Cohen, 1998). This paradigm allows researchers to introduce conflicts between multisensory cues and thus to investigate the effect of different cues on self-representation. In the conventional RHI paradigm, an artificial hand is placed next to a participant's hidden hand. When both hands are stroked at the same time, this can induce an experience of embodiment such that the participant feels that the artificial hand is part of their own body. Researchers have manipulated cues such as the synchrony of tactile and visual stimulation or the form of the artificial hand to investigate which signals are important drivers of the sense of embodiment. Embodiment in this paradigm is typically measured by asking participants to report their subjective experience related to body ownership, agency, and the perceived location of their limb (Tsakiris and Haggard, 2005; Longo et al., 2008b), or with action-oriented measures that involve decisions, actions or physiological reactions to stimuli near the body (Armel and Ramachandran, 2003; Aspell et al., 2009; Zopf et al., 2010, 2011, 2013).

### Agency cues

Although agency has sometimes been considered an element of embodiment, there has also been considerable research investigating the sense of agency as an independent construct, using a variety of experimental designs. In some of these designs, specific experimental cue manipulations changed the kind of cognitive attributions or sensory predictions participants made about their own or others' actions. For example, in Wegner and Wheatley's (1999) “I-Spy” task, false audio commentaries describing the motion of a mouse cursor displayed on a computer screen led participants to misattribute their own thoughts as the cause of the actions they observed, even though these actions were actually externally generated. Conversely, in the Blakemore et al. (2000) study of tickle responses, a mechanical device was used to introduce a temporal delay between participants' tickling actions and the tactile stimulus from those actions. This manipulation interfered with participants' sensory predictions regarding the outcome of their intended actions and led them to experience these self-generated movements as if they had been externally generated. In another line of research, hypnotic suggestions have been shown to induce significant changes in the way that susceptible participants generate and monitor actions, leading to marked alterations to the sense of agency (Polito et al., 2014). Sense of agency has been measured in a variety of ways including explicit ratings of first-person experience (Bowers, 1981; Polito et al., 2014, 2015) and indirect, implicit measures such as intentional binding, which uses participants' time judgments regarding causal actions in a behavioral task as a proxy for agency (Haggard et al., 2002).

### Presence cues

Research into cues that influence the sense of presence has typically taken two forms. First, some studies have investigated how the experience of being present in a virtual environment is affected by the technical capacity of the VR hardware to deliver realistic multisensory cues. These studies include the impact on presence of: head tracking and provision of stereoscopic 3D cues (Hendrix and Barfield, 1995; Barfield et al., 1999); VR display resolution and refresh rate (Barfield and Hendrix, 1995); latency between head movement and VR display updating, and the inclusion of haptic feedback (Sanchez-Vives and Slater, 2005). Such studies of presence are also clearly important for the consumer VR industry. For example, the *Oculus Best Practices* (Oculus, 2016) emphasizes the importance of achieving < 20 ms latency between head movements and corresponding screen updates, in addition to maximizing screen refresh rate, to avoid negative impacts on user comfort and presence.

The second area of presence research aims to identify the specific cues and content within virtual environments that lead to increased presence. For example, Slater et al. (2009a) and Yu et al. (2012) investigated the extent to which visual realism of the virtual environment affects presence. Their work concluded that it was the dynamic nature of shadows and reflections in response to events rather than mere lighting and reflection quality that primarily drives a sense of presence (Yu et al., 2012).

Post-experiment subjective questionnaires with explicit rating scales are a primary tool for measuring presence. Some researchers, however, have sought to measure presence more objectively by employing implicit behavioral and physiological reactions such as measuring changes in heart rate (Sanchez-Vives and Slater, 2005).

### Investigating self-representation using the virtual hand illusion

These separate lines of research into embodiment, agency, and presence have found that each component involves the integration of multiple cues both within and across sensory modalities. This suggests that these components may partly rely on similar underlying mechanisms. However, the degree to which this may be true remains unclear. Some authors, for example, have suggested that components such as ownership and agency may directly influence each other (Tsakiris et al., 2006; Morgan, 2015). Alternatively, these components may simply rely on similar cues (Synofzik et al., 2008; Zhang et al., 2014). As a step toward addressing this issue, in this study we systematically manipulated a range of cues and studied their effect on the different components of self within a single experimental context. The context we chose is a variant of the RHI paradigm. Specifically, we implemented a new VR version of this paradigm—the Virtual Hand Illusion (VHI, Slater et al., 2008).

RHI-type paradigms commonly involve manipulations of various cues across the modalities of vision, touch, and proprioception. The experience of embodiment in the RHI results from the integration of multiple sensory cues that could plausibly provide body relevant information (Tsakiris, 2010; Apps and Tsakiris, 2014; Blanke et al., 2015; Kilteni et al., 2015).

Three key cues that have been found to modulate embodiment in the RHI are: (a) *visual-form plausibility* (hereafter FORM), how realistically the experimental stimulus resembles a body (Tsakiris and Haggard, 2005; Tsakiris et al., 2010); (b) *visual–tactile synchrony* (hereafter TOUCH), the consistency in timing between tactile stimulation applied to a participant's own hand and the visual representation of stimulation applied to the rubber hand (Botvinick and Cohen, 1998); and (c) *visual–proprioceptive spatial position offset* (hereafter OFFSET), the distance between the proprioceptively localized real hand and the visually localized rubber hand. A number of researchers (e.g., Tsakiris, 2010; Blanke et al., 2015) have claimed that introducing discrepancies to these cues can constrain the inclusion of the artificial hand as a part of bodily self-representation.

However, three findings in the literature indicate that introducing discrepancies to these cues may not always act as hard limits on embodiment. First, placing the artificial hand far from the actual hand (i.e., introducing a large spatial OFFSET between vision and proprioception) does not always affect the RHI, especially when the viewed hand is placed near the trunk (Zopf et al., 2010; Preston, 2013). Second, in a recent VHI study where participants were able to move the virtual limb, embodiment effects were found even when the visual form of the target stimulus was a balloon or square rather than a virtual hand (Ma and Hommel, 2015). It is, however, unclear if this is also the case in a passive version of the illusion. Third, previous data suggest that TOUCH might have its strongest effect when there is a spatial offset between the hands (Zopf et al., 2010).

To the best of our knowledge, no previous study has systematically manipulated all three cues (FORM, TOUCH, and OFFSET) within a single experimental context. For each cue, these manipulations involve altering the degree of alignment between the presented visual feedback and other sources of information regarding the body. For example, the effect of FORM can be tested by comparing a condition where the virtual hand form is congruent with that of the real hand, vs. a condition where the virtual form is not at all hand-like, such as presenting a simple block or sphere. Similarly, the effect of TOUCH can be tested by altering the synchronization between seen and felt touch stimuli. Finally, the effect of OFFSET can be tested by introducing a discrepancy between the visually presented and felt real hand position. Exploring each of these cues within a single experimental context permits examination of main effects and interactions, both of which are critically important for determining the relative importance of the individual cues.

In this study, we simultaneously manipulated FORM, TOUCH, and OFFSET to investigate their effects and interactions on embodiment as well as other aspects of self-representation such as the sense of agency and presence in an RHI-type paradigm. For FORM and OFFSET we used two conditions each, a congruent and an incongruent condition. For TOUCH, in addition to the commonly employed synchronous and asynchronous conditions, we also included a “no-touch” control condition in which no active tactile stimulation is delivered, (passive touch still occurs via the hand resting on the table). Little is known about how the mere occurrence of a touch might influence embodiment, since only a few previous studies have employed a no-touch condition (Longo et al., 2008a; Rohde et al., 2011).

### Rationale for using the virtual hand illusion

In this study, we employed a VHI paradigm, which is an adaptation of the RHI to a virtual environment (Slater et al., 2008). The VHI paradigm offers several methodological advantages compared to the standard RHI. Computer simulations allow a high level of experimental control, continuity and precise repeatability for stimulus presentation. Using a virtual hand makes it easy to carefully manipulate many aspects of visual form, for example, changing hand shape while keeping skin texture constant. Also, the contextual break that results from placing a fake hand model in a real world context can be avoided by using VR to seamlessly present a virtual hand model in a similarly virtual environment. Furthermore, the VHI setup allows for consistent matching between the presentation times of tactile and visual stimuli, providing a greater level of temporal reliability compared to the experimenter-generated manual brushing commonly employed in the RHI. Finally, the presentation of visual stimuli is not restricted due to physical interference from the artificial limb. Instead, stimuli can be presented anywhere within the virtual environment making it much easier to achieve true overlap (no apparent spatial OFFSET) between virtual and actual body parts.

A former disadvantage of the VHI compared to the RHI has been the challenge, time and cost of creating virtual environments and acquiring VR equipment. Until very recently, VR technology was limited to specialty research and niche training applications such as flight simulator training or other military applications, but this is no longer true. Affordable, high quality VR head-mounted displays (HMDs) such as the Oculus Rift are now commercially available. The confluence of consumer VR with the mainstreaming of video-game and other computer-generated video media also means that powerful and easy-to-use desktop 3D environment creation software is now readily available, supported by online marketplaces with large user communities of enthusiasts, graphic artists, and developers. Consequently, researchers can now design and implement experiments using VR with relative ease and at reasonable costs. In light of this, a supplementary motivation of this study was to develop and demonstrate the viability of conducting cognitive science research using consumer-grade VR technology.

### Investigating the viability of consumer VR for research

Despite the advantages and ease of creating virtual environments, there are a variety of non-trivial sizing and positioning challenges to achieve high congruency between the virtual and the real. A good match means that virtual features such as chairs, tables, hands, and viewing position are visually at the same scale, position and orientation as corresponding real features. Failure to carefully solve these challenges may introduce uncontrolled spatial and sizing conflicts between visual and proprioceptive feedback, which could impact experiment results and obscure analysis of the specific cue manipulations we wished to perform. In this study we therefore also aimed to demonstrate clear methods for matching the real and virtual worlds using consumer VR.

### Summary of aims and hypotheses

To summarize, in this study we investigate three distinct components of self-representation—embodiment, agency and presence—within a single experimental context. Our first aim was to systematically investigate how FORM, TOUCH, and OFFSET influence these components of self-representation. To measure changes in self-representation we employed rating scales and items that have previously been used in embodiment (Longo et al., 2008b), agency (Polito et al., 2013), and presence (Sanchez-Vives and Slater, 2005) research. Our second aim was to demonstrate that scientific research on self-representation can be conducted successfully using consumer-grade VR technology.

We formulated five specific hypotheses concerning the influence of FORM, TOUCH, and OFFSET cues on self-representation. First, based on previous work (Tsakiris and Haggard, 2005; Tsakiris et al., 2010), we predicted that using an incongruent FORM in our VHI paradigm would negatively impact embodiment ratings compared to the congruent FORM. Second, we predicted that changes in TOUCH would influence embodiment measures for both plausible visual forms (i.e., hands), and for implausible visual forms (i.e., simple geometric volumes that are not hand-shaped, see Ma and Hommel, 2015). In other words, we expected a main effect of TOUCH but no interaction of TOUCH and FORM. Third, based on previous findings (Zopf et al., 2010; Preston, 2013), we expected that spatial OFFSET would not have a strong overall effect on embodiment. Fourth, based on earlier work showing an increased effect of TOUCH on embodiment when a rubber hand is displaced from a participant's actual hand (Zopf et al., 2010), we predicted that TOUCH would be most important when there is a spatial discrepancy between vision and proprioception. That is, we expected an interaction between TOUCH and OFFSET. Our fifth hypothesis concerned the sense of agency. We expected that the occurrence of touch would influence sense of agency since tactile signals indicate that an action is occurring in the external environment, such as contacting a surface while reaching for an object. As agency is robust to noisy sensory signals (Moore and Fletcher, 2012), we specifically expected that the mere occurrence of tactile feedback, rather than visual-tactile synchrony *per se*, would influence agency ratings. Since FORM and OFFSET cues do not provide obvious indications of action, we did not expect these manipulations to influence sense of agency ratings. We did not have strong predictions regarding presence, as the effect of these cues has received relatively little attention in the relevant literature.

## Methods

### Participants

We tested 50 participants who either received course credit for participation or payment of $15. Twenty-five participants (16 female, 21 right handed, mean age 21 years, range 18–34 years) completed the experiment with a zero spatial OFFSET condition and 25 participants (14 female, 25 right-handed, mean age 20 years, range 18–32 years) completed the experiment with a non-zero spatial OFFSET condition. This research was conducted in accordance with the ethical standards laid down in the 1964 Declaration of Helsinki and was approved by the Macquarie University Ethics Review Committee (Human Research). Informed consent was obtained from all participants.

### Equipment

**Figure 1:**
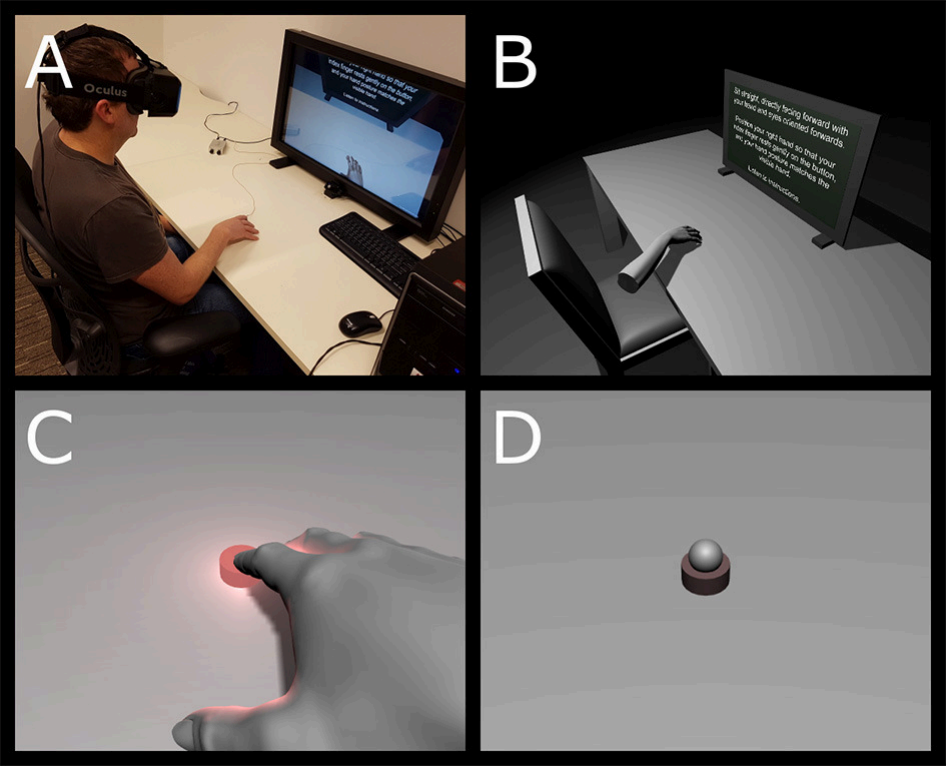
**(A)** Experimental setup, with the tactor positioned beneath the right index finger. The monitor offers a 2D depiction of the participant's view for the experimenter to observe. **(B)** An overhead view of the entire virtual environment (i.e., not from the participant's viewpoint). **(C)** Virtual hand/finger placement, also depicting the visual feedback corresponding to a touch. Dynamic illumination effects such as the light flash reflection from the hand and table are visible. **(D)** A close up of the virtual button in the “no touch” condition with the incongruent FORM (a sphere).

The primary device for delivery of all VR experiments was the Oculus Rift Development Kit 2^1^ (DK2), depicted in Figure 1A. The DK2 is a HMD with positional camera tracking system that allows six degrees of freedom head tracking (head rotation and translation). We chose PC hardware sufficient to maintain the visual frame rate at the maximum DK2 display refresh rate of 75 Hz at the native DK2 display resolution (960^*^1080 pixels per eye), with no transient drops in frame rate, frame skipping, or latency spikes. Full specifications for the DK2 and PC hardware are included in Appendices A and B in Supplementary Material.

Tactile stimuli were delivered via a vibrating tactor device placed beneath the participant's index finger. Tactor oscillations were driven via 200 Hz sinewave audio outputs from the PC's audio processing card. Technical details for the tactor and PC audio processing are provided in Appendix B in Supplementary Material.

### 3D environment and software

We used the Unity^2^ 3D videogame engine, version 5.1.2f1 (64-bit) software to construct the 3D environment. The environment was a simple monochrome space with no complex graphical textures (see Figures 1B,C). The virtual space resembled the actual lab environment and consisted of gray floor, desk, chair, and a virtual computer screen that were illuminated via a virtual light source from above. There were no walls and illumination faded to black if the participant looked into the distance.

Our experiment maintained dynamic illumination which has been shown to increase the plausibility of the VR experience (Khanna et al., 2006; Slater et al., 2009a; Yu et al., 2012) and is readily achievable with the Unity 5 engine (including soft-edged shadows and real-time light from the flashing virtual button cast onto nearby objects such as the hand, table and virtual monitor). Virtual objects used in the scene, including furniture and the hand model, were either created directly within Unity (simple geometric solids) or acquired at no cost online. We used a consistent, gender neutral arm model for all participants^3^.

### Calibrating the virtual environment

To ensure that the apparent size and position of virtual objects matched the real environment, and to also calibrate the participant's virtual viewpoint to their real-world viewpoint, we adopted the following procedures.

#### Similar plain appearance for real and virtual objects

We measured the dimensions and placement of real-world objects involved in the experiment, and kept the general appearance of the real desk and walls unadorned and featureless. These object dimensions and similarly plain appearance were reproduced in the virtual environment.

#### Benchmarking virtual space and size

Aligning the virtual and real environments involved calibration along two separate factors. First, units of distance in the virtual environment were benchmarked against real distances in the physical workspace environment to ensure a close match. We assessed this by moving the HMD 50 cm along the desk within view of the position-tracking camera, and noting the real-time movement of the corresponding viewpoint in the virtual environment. After repeated testing, we established that (at least for our setup) 100 cm in the real workspace environment corresponded to 0.96 distance units in Unity. This ratio was used in creating and positioning virtual objects (e.g., the desk and chair) to achieve close alignment between the physical and virtual environments.

#### Calibrating scaling for each participant

In VR, depth perception is achieved via binocular stereopsis by presenting an offset camera view to each eye. The distance between these two virtual cameras is directly correlated to what the participant perceives as their own size in the virtual environment, and therefore affects the participant's sense of the relative scale of all virtual objects and distances. So that each participant perceived the virtual world with the same sense of scale as they do in the real world, we measured the distance between each participant's pupils (inter-pupillary distance or IPD), and set a corresponding separation between the virtual camera view for each eye. This separation is readily manipulated by entering the participant's IPD directly into the Oculus Rift DK2 configuration utility^4^.

IPD was initially measured using a utility provided with the Oculus runtime. However, this procedure proved time consuming and occasionally produced obviously incorrect measurements, and was abandoned after 18 participants. For the remaining participants, we instead measured IPD using a ruler positioned against the participant's nose. When compared over several tests the two measurement techniques were within ±2 mm of one another.

#### Positioning the virtual viewpoint to match the real-world viewpoint

We used a small, custom-made HMD mount to locate the HMD in a preset real-world position and orientation prior to each experiment, within view of the positional-tracking camera. Once the HMD's real-world position/orientation were fixed by placing it in the mount, the experimenter could shift the virtual viewpoint to align with the HMD's real world position/orientation with a single key press. Following this, the HMD could then be moved around and placed on the participant's head. The positional-tracking camera would maintain an accurate record of its real-world location, and adjust the virtual viewpoint accordingly in real time to maintain alignment with the participant's head movements. This calibration step was repeated after every experiment trial. The HMD mount was removed from the setup while running the experiment trial itself.

#### Appropriately situating the participant's real hand

As described below, the experiment design required appropriate positioning and posture of the participant's real right hand to enable a match or mismatch (depending on the OFFSET condition) between the proprioceptive feedback from the real hand and visual feedback from the virtual hand. The location of the participant's real hand and index finger were controlled by adhering the tactor to specific physical locations on the desk for each of the OFFSET conditions and instructing participants to place the tip of their index finger on the tactor. Participants were also instructed to align their real hand posture to that of the virtual hand for trials involving the hand FORM condition.

### Multisensory touch stimulation in VR

Visual–tactile feedback to the participant consisted of a periodic vibration delivered by a tactor positioned beneath the participant's right index finger. Participants wore headphones to mute both the audible noise resulting from the vibration of the tactor unit, and any other unwanted environmental noise.

### Self-representation rating scales

To measure embodiment, agency, and presence, we employed three sets of rating scales.

#### Embodiment rating scales (Botvinick and Cohen, 1998; longo et al., 2008b)

We used the 10 embodiment rating scale items from Longo et al. (2008b) as well as the item “It seemed like I was feeling the touch in the location where I saw the rubber hand being touched,” which is often included in RHI studies and positively rated in synchronous conditions (Botvinick and Cohen, 1998). As the viewed stimulus varied across trials in this task (i.e., a hand or a sphere), the wording of items in this questionnaire was modified to refer to “the target.” Based on the findings of Longo et al. we further divided these 11 items into three different subcomponents (see Table 1 for details): *embodiment–ownership* (for example, “It seemed like the target belonged to me”), *embodiment–location* (for example, “It seemed like the target was in the location where my hand was”), and *embodiment–agency* (“It seemed like I was in control of the target”). For each item participants rated their level of agreement on a 7-point Likert scale from “strongly disagree” to “strongly agree.” In the “no touch” condition, the items referring to touch experience (8 and 9) were not presented. We computed average embodiment component scores for ownership, location and agency for each participant and condition.

**Table 1:** **Embodiment rating scale (Based on Longo et al., 2008b)**.

	**Item**	**Subscale**
1.	It seemed like I was looking directly at my own hand rather than the target	Ownership
2.	It seemed like the target began to resemble my real hand	Ownership
3.	It seemed like the target belonged to me	Ownership
4.	It seemed like the target was my hand	Ownership
5.	It seemed like the target was part of my body	Ownership
6.	It seemed like my hand was in the location where the target was	Location
7.	It seemed like the target was in the location where my hand was	Location
8.^*^	It seemed like the touch I felt was caused by the button flash at the target	Location
9.^*^	It seemed like I was feeling the touch in the location where I saw the target being touched	Location
10.	It seemed like I could have moved the target if I had wanted.	Agency
11.	It seemed like I was in control of the target	Agency

* *These items not included in “no-touch” conditions*.

#### The sense of agency rating scale (SOARS; Polito et al., 2013)

The SOARS is a 10-item scale that measures subjective alterations to the sense of agency related to some specific experience. Participants were instructed to think of the preceding experimental task and to rate their level of agreement with a series of statements on a 7-point Likert scale from “strongly disagree” to “strongly agree.” The scale has two factors: (1) *involuntariness*, with items such as “I felt that my experiences and actions were not caused by me,” which represent a subjectively-experienced reduction in control over one's own actions; and (2) *effortlessness*, with items such as “My experiences and actions occurred effortlessly,” which represent a subjectively-experienced increase in the ease and automaticity with which actions occur. Although, the SOARS was originally developed for use in hypnosis, we used a modified, general form with slight edits to the wording of three items (#1, #4, and #10), which is applicable in any context (Table 2).

**Table 2:** **General form of the Sense of Agency Rating Scale (Polito et al., 2013)**.

	**Item**	**Subscale**
1.^*^	Doing what I was meant to was hard	Effortlessness
2.^*^	I chose how to respond	Involuntariness
3.^*^	My experiences and actions felt self-generated	Involuntariness
4.	I went along with my experiences freely	Effortlessness
5.^*^	My experiences and actions were under my control	Involuntariness
6.	I felt that my experiences and actions were not caused by me	Involuntariness
7.	My experiences and actions occurred effortlessly	Effortlessness
8.	I was mostly absorbed in what was going on	Effortlessness
9.	My responses were involuntarily	Involuntariness
10.^*^	I was reluctant to go along with my experiences	Effortlessness

* *These items are reverse scored*.

#### Presence rating items (Sanchez-Vives and Slater, 2005)

Participants rated three presence items on a 7-point Likert scale: (1) “To what extent did you have a sense of being in the virtual environment,” rated from “not at all” to “very much so”; (2) “To what extent were there times during the experience when the virtual environment became “reality” for you, and you almost forgot about the “real world” of the laboratory in which the whole experience was really taking place?,” rated from “never” to “almost all the time”; and (3) “When you think back to your experience, do you think of the virtual environment more as *images* that you saw, or more as *somewhere that you visited*?,” rated from “only images that I saw” to “somewhere that I visited.” These items are reported by Sanchez-Vives and Slater (2005) as representative items for assessing alterations in presence. Although the descriptive poles of the Likert scale differ across items, in all cases a score of 1 represents no presence, whereas a score of 7 represents complete presence.

### Experimental design

The experiment included FORM, TOUCH, and OFFSET manipulations. We implemented two within-subject FORM conditions (see Figures 1C,D): (a) a congruent feedback condition involving the presentation of a realistically depicted virtual hand and forearm shape, in which the index finger was positioned on top of a realistically depicted virtual tactor on a virtual table. The virtual hand had smooth, unmarked texturing and light gray coloring; and (b) an incongruent hand feedback condition involving the presentation of a smooth, unmarked gray spherical object (~1.8 cm in apparent diameter), appearing atop the virtual tactor (Figure 1D) in the same position as the tip of the participant's real index finger. The virtual tactor could be presented in one of two states: either visually vibrating and glowing bright red (an ON state corresponding to the delivery of tactile feedback) or completely motionless with a dull dark red color (an OFF state corresponding to the absence of tactile feedback).

For TOUCH manipulations, there were three within-subject conditions: (a) synchronous touch—visual and tactile stimulation were initiated at the same time and presented for 300 ms every 1000 ms; (b) asynchronous touch—the tactile stimulation was identical to the synchronous case, while the visual flash/vibration was initiated at random intervals between 500 and 1500 ms after the tactile feedback; and (c) no touch—no visual or tactile vibration stimulation was given.

Although they were initiated simultaneously in code, we tested the system delay timing using a 240 frames/second audio-visual camera. We recorded onset of visual feedback through the HMD lenses and onset of tactor vibrations by increasing vibration amplitude sufficiently to produce an audible sound that could be recorded by the camera's microphone. In analysing 25 recorded tactor vibrations, we found that there was a mean delay of 226 ms (*SD* 13 ms) between the visual flash and the tactor vibration. This delay meant that the visual and tactile stimulation was not completely synchronous in the synchronous condition. However, small delays due to human error would also be expected in research employing manual brush stroking. Previous RHI research found no difference between delays as long as 300 ms compared to smaller delays for inducing changes in embodiment (Shimada et al., 2009). In addition, the low variability in delay magnitude from vibration to vibration means that better consistency for this experiment is likely than for RHI research employing manual brush stroking.

Finally, we had two between-subjects OFFSET conditions: (1) 0 cm spatial offset, with the participant's real hand positioned along the body midline, in the same apparent position as the virtual hand, and (2) 30 cm offset, with the participant's real right hand positioned 30 cm to the right of the body midline, while the virtual hand (or sphere) position was maintained in the exact position and orientation as in the 0 cm offset condition. OFFSET was investigated as a between subject condition partly to keep the experiment duration reasonable (a third within-subject condition would double the experiment duration), but also to minimize the potential for leading the participant: for FORM and TOUCH manipulations, participants can receive identical instructions and experiment setup is unchanged. Changing the OFFSET condition requires moving the placement of the tactor on the desk and moving the participant's hand. This may have signaled a change to the participant and influenced their responses.

Each participant was tested in one of these two OFFSET conditions and completed all FORM and TOUCH conditions in a 2-by-3 factorial design—six trials per participant. There are 720 possible orderings of the six conditions. Since we only used 25 orderings, we adopted a pseudo-random ordering selection for each participant. This involved randomly selecting an order from the 720 possible orderings for a participant, and then using the reverse of this for the next participant. The same orderings were used for the 25 participants tested with the 0 cm offset condition as for the 25 participants tested with the 30 cm offset condition.

### Procedure

Following IPD measurement and positional calibration, the participant was seated and the HMD placed on the participant's head. We ensured that the positional tracking camera did not lose view of the HMD while this was done, to maintain the virtual- to real-environment match. The experimenter then assisted the participant in adjusting their real chair so that they were sitting with their torso a few centimeters from the edge of the desk, and central to the scene. In discussion with the participant, the experimenter fitted the straps and HMD position to ensure optimal focus. Participants were asked to adjust the HMD on their heads until they had good focus in the center of their vision. Participants were instructed to keep their left arm in their lap where it would be obscured from view by the desk (this ensured consistency with the virtual environment where no left arm was visible), to sit up straight, and avoid leaning back or rotating the chair position. Participants were otherwise free to look around the scene or to lean in to view objects in the scene more closely.

Participants began each trial viewing the desk scene without any local tactile stimulus, with instructions displayed on the virtual monitor. While viewing this scene, the experimenter would ensure the participant was correctly positioned and also place headphones on the participant. At the commencement of each of the six experiment trials, the participant would first see 15 s of darkness, before again viewing the same desk scene for the experiment trial proper. Participants would view the target and experience visual–tactile stimulation (or not, in the case of the “no touch” trials) for a 1-min duration. During this time, participants were instructed to keep their arm still, but were free to move their head as desired to view the target from any angle. Following each trial, participants were prompted to remove the HMD and complete a questionnaire.

At the beginning of responding to the set of rating items the participant was told that the target referred to either the “gray hand” or the “gray sphere” as appropriate. In order to minimize stereotyped responses, the embodiment, agency, and presence rating item sets were presented in random order. Furthermore, the order of items within each set was randomized. The ratings were presented using Qualtrics Survey Software (www.qualtrics.com) on a separate laptop.

Positional calibration using the HMD mount was performed at the start of each experiment trial. Each of the six variations was prepared in separate executable files. The experiment duration, including IPD measurement, instructions, and the six trials, was typically 30–45 min.

## Results

To investigate the effects of FORM, TOUCH, and OFFSET, we entered all rating scale means (*embodiment–ownership, embodiment–location, embodiment–agency, presence, involuntariness, effortlessness*) into a multivariate analysis of variance (MANOVA) with the within-subject factors FORM (hand, sphere) and TOUCH (synchronous, asynchronous, no touch stimulation), and the between-subject factor OFFSET (0 cm spatial offset, 30 cm spatial offset). To further investigate the effect of different cues on each self-representation scale separately, we conducted individual ANOVAs with the factors FORM, TOUCH, and OFFSET. We found non-normal rating response distributions for some rating scales (Shapiro–Wilk-tests). However, ANOVA are robust also for non-normally distributed data when the sample size is equal (Field, 2009).

**Figure 2:**
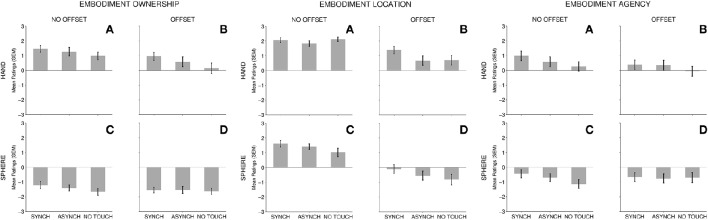
*****Embodiment*** mean rating scale results**. For each subscale, TOUCH conditions (Synchronous, Asynchronous, No Touch) are depicted for all FORM and OFFSET conditions: **(A)** No Offset—Hand; **(B)** Offset—Hand; **(C)** No Offset—Sphere; and **(D)** Offset—Sphere. Error bars present the standard error of the mean.

**Figure 3:**
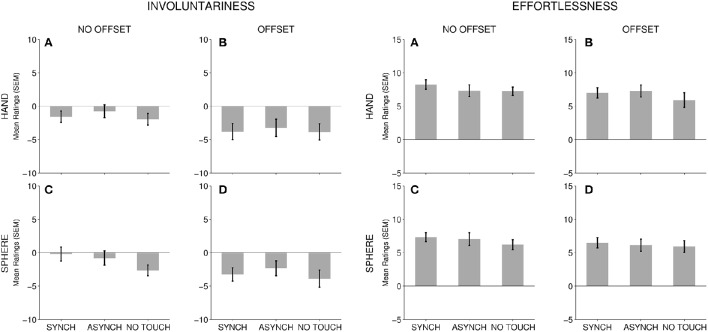
*****Agency*** mean rating scale results**. For each subscale, TOUCH conditions (Synchronous, Asynchronous, No Touch) are depicted for all FORM and OFFSET conditions: **(A)** No Offset—Hand; **(B)** Offset—Hand; **(C)** No Offset—Sphere; and **(D)** Offset—Sphere. Error bars present the standard error of the mean.

**Figure 4:**
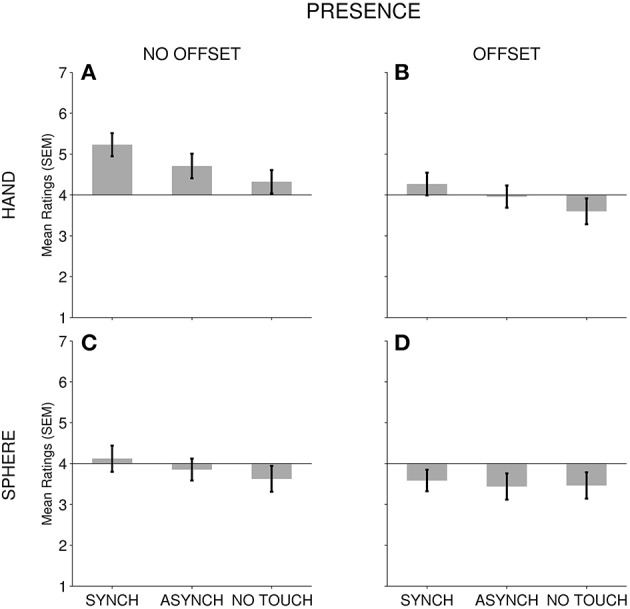
*****Presence*** mean rating scale results**. For each subscale, TOUCH conditions (Synchronous, Asynchronous, No Touch) are depicted for all FORM and OFFSET conditions: **(A)** No Offset—Hand; **(B)** Offset—Hand; **(C)** No Offset—Sphere; and **(D)** Offset—Sphere. Error bars present the standard error of the mean.

For FORM, there was a multivariate main effect across all rating scales [Pillai's trace, *V* = 0.756, *F*_(6, 43)_ = 22.25, *p* < 0.001, η_*p*_^2^ = 0.756]. ANOVA for each rating scale separately showed that viewing a hand resulted in significantly higher mean values compared to viewing a sphere for *embodiment–ownership, embodiment–agency, embodiment–location, presence* and *effortlessness*, but not *involuntariness* (see Table 3 for statistics and Figures 2–4).

**Table 3:** **Statistics (***F*** and ***p***-values and effect sizes) for Multivariate and Univariate Main effects for FORM, TOUCH, and OFFSET**.

	**FORM**			**TOUCH**			**OFFSET**		
	***F***	***P***	**η_*p*_^2^**	***F***	***p***	**η_*p*_^2^**	***F***	***p***	**η_*p*_^2^**
*Multivariate*	**22.25**	**<0.001**	**0.756**	**2.50**	**0.016**	**0.448**	**6.01**	**<0.001**	**0.456**
Ownership	**142.17**	**<0.001**	**0.748**	**8.28**	**<0.001**	**0.147**	2.76	0.103	0.054
Location	**44.70**	**<0.001**	**0.482**	**8.37**	**<0.001**	**0.148**	**28.77**	**<0.001**	**0.375**
Agency	**34.60**	**<0.001**	**0.419**	**5.19**	**0.007**	**0.098**	0.26	0.615	0.005
Involuntariness	0.60	0.443	0.012	**3.79**	**0.026**	**0.073**	2.86	0.097	0.056
Effortlessness	**5.24**	**0.027**	**0.098**	2.79	0.066	0.055	0.65	0.425	0.013
Presence	**29.81**	**<0.001**	**0.383**	**9.81**	**<0.001**	**0.170**	2.85	0.098	0.056

*Bold text indicates a significant result*.

We found a multivariate main effect of TOUCH [Pillai's trace, *V* = 0.448, *F*_(6, 43)_ = 2.50, *p* = 0.016, η_*p*_^2^ = 0.448]. ANOVA for each variable separately showed that TOUCH was significant for *embodiment–ownership, embodiment–agency, embodiment–location, presence*, and *involuntariness*. We found a trend for *effortlessness* (Table 3). We also used planned, simple contrasts to directly compare the effect of synchronous and asynchronous touch (effect of *touch synchrony*) as well as between synchronous and asynchronous touch combined and compared to no touch (effect of *touch occurrence*). We found a significant effect of touch synchrony for *embodiment–location* and *presence*, such that synchronous touch led to higher self-representation ratings [*embodiment–location*: *F*_(1, 48)_ = 8.03, *p* < 0.001, η_*p*_^2^ = 0.269; *presence*: *F*_(1, 48)_ = 8.88, *p* = 0.005, η_*p*_^2^ = 0.156]. Surprisingly, there was only a trend for *embodiment–ownership* [*F*_(1, 48)_ = 3.93, *p* = 0.053, η_*p*_^2^ = 0.076]. There was no effect of touch synchrony for any of the agency measures [*embodiment–agency*: *F*_(1, 48)_ = 2.07, *p* = 0.156, η_*p*_^2^ = 0.041, *involuntariness*: *F*_(1, 48)_ = 0.802, *p* = 0.375, η_*p*_^2^ = 0.016, *effortlessness*: *F*_(1, 48)_ = 0.802, *p* = 0.375, η_*p*_^2^ = 0.016]. In contrast, we found a significant effect of touch occurrence for all scales [*embodiment–ownership*: *F*_(1, 48)_ = 10.71, *p* = 0.002, η_*p*_^2^ = 0.182, *embodiment–agency: F*_(1, 48)_ = 7.64, *p* = 0.008, η_*p*_^2^ = 0.137, *embodiment–location: F*_(1, 48)_ = 4.71, *p* = 0.035, η_*p*_^2^ = 0.089, *involuntariness*: *F*_(1, 48)_ = 4.06, *p* = 0.050, η_*p*_^2^ = 0.078, *effortlessness: F*_(1, 48)_ = 6.67, *p* = 0.013, η_*p*_^2^ = 0.122, and *presence: F*_(1, 48)_ = 10.31, *p* = 0.002, η_*p*_^2^ = 0.177], such that each of these ratings were higher for touch compared to no-touch conditions. To summarize, for *embodiment–ownership*, e*mbodiment–location*, and *presence*, touch synchrony, and touch occurrence were both significant factors. In contrast, agency measures (*embodiment–agency, effortlessness*, and *involuntariness*) were sensitive only to touch occurrence (a trend only for *involuntariness*). We found no significant multivariate or univariate interactions between FORM and TOUCH, or between TOUCH and OFFSET (Table 4). This indicates that the effect of TOUCH does not differ for hand and sphere forms, or when the virtual hand location is displaced relative to the actual hand.

**Table 4:** **Statistics (***F*** and ***p***-values and effect sizes) for Multivariate and Univariate Interaction effects for FORM, TOUCH, and OFFSET**.

	**FORM × TOUCH**			**FORM × OFFSET**			**TOUCH × OFFSET**			**FORM × TOUCH × OFFSET**		
	***F***	***p***	**η_*p*_^2^**	***F***	***p***	**η_*p*_^2^**	***F***	***p***	**η_*p*_^2^**	***F***	***p***	**η_*p*_^2^**
*Multivariate*	1.21	0.311	0.282	**3.64**	**0.005**	**0.337**	1.92	0.063	0.384	0.47	0.920	0.132
Ownership	1.57	0.214	0.032	1.78	0.188	0.036	0.01	0.994	0	1.32	0.273	0.027
Location	2.13	0.124	0.043	**5.89**	**0.019**	**0.109**	1.67	0.194	0.034	0.83	0.441	0.017
Agency	0.37	0.692	0.008	1.27	0.265	0.026	1.27	0.287	0.026	0.65	0.527	0.013
Involuntariness	1.07	0.349	0.022	0.10	0.757	0.002	0.64	0.533	0.013	0.55	0.581	0.011
Effortlessness	0.05	0.950	0.001	0.11	0.745	0.002	0.24	0.786	0.005	0.93	0.399	0.019
Presence	2.08	0.131	0.042	3.27	0.077	0.064	0.77	0.466	0.016	0.12	0.890	0.002

*Bold text indicates a significant result*.

For OFFSET, we found a multivariate main effect [Pillai's trace, *V* = 0.456, *F*_(6, 43)_ = 6.01, *p* < 0.001, η_*p*_^2^ = 0.456]. Individual ANOVAs revealed an unsurprising main effect of *embodiment–location*, such that participants gave higher ratings in the no spatial offset condition compared to the spatial offset condition. No significant OFFSET effect was found for any of the other scales (Table 4).

Furthermore, there was a multivariate interaction of FORM-by-OFFSET, indicating that the overall effect of FORM on our measures of self-representation depended on whether the apparent location of the virtual hand was displaced from the location of the actual hand [Pillai's trace, *V* = 0.337, *F*_(6, 43)_ = 3.64, *p* = 0.005, η_*p*_^2^ = 0.337]. Univariate ANOVAs for each rating scale separately showed this interaction to be significant for *embodiment–location* only (see Table 4 for statistics). As can be seen in Figure 2 (“Embodiment Location” panel), the difference between viewing a hand and viewing a sphere was relatively small in the no spatial offset condition, whereas a change in FORM had a much greater effect when there was a spatial offset. Viewing a sphere in the spatial offset condition led to negative *embodiment–location* scores, whereas *embodiment–location* ratings were positive when viewing a hand, regardless of OFFSET. We did not find a FORM-by-OFFSET interaction for any of the other variables (Table 4).

There was no multivariate or univariate 3-way interaction of FORM, TOUCH, and OFFSET (Table 4).

## Discussion

This study utilized a novel consumer grade VR system to test the impacts of FORM, TOUCH and OFFSET on self-representation in a Virtual Hand Illusion paradigm. We used multiple measures including an embodiment scale commonly used in RHI studies (Longo et al., 2008b); the Sense of Agency Rating Scale (SOARS), which has previously been used to assess alterations to feelings of agency in studies of self-generated actions in hypnotic and clinical contexts (Polito et al., 2013, 2014, 2015); and rating scale items for presence typically used in studies of VR experiences (Sanchez-Vives and Slater, 2005). We found multivariate effects of FORM, TOUCH, and OFFSET across all measures, confirming that each of these cues has a broad influence on self-representation. We tested five specific predictions about the effects of our experimental manipulations.

### Hypothesis one: the effect of form on embodiment

As expected, we found that a congruent visual representation of the hand led to higher scores on all embodiment subscales compared to the incongruent spherical cursor representation. Previous studies have shown that FORM congruency is an important factor in the traditional RHI setup (Tsakiris and Haggard, 2005; Tsakiris et al., 2010), and these results also confirm this for the VHI. The FORM resemblance of visual body representations thus seems an important driver of embodiment.

### Hypothesis two: the effect of touch on embodiment

In line with our expectations the type of touch influenced all embodiment subscales. This was not modulated by the FORM of the target. This is consistent with findings from a recent study by Ma and Hommel (2015), in which artificial objects on a screen changed either synchronously or asynchronously with the participant's hand movements. Those authors found that movement feedback synchrony modulated embodiment ratings even for non-body objects. Here, we extend this finding and report that FORM does not seem to significantly limit the effect of touch synchrony, even with a static target. We found no evidence that the effect of multimodal synchrony on self-representation is limited or constrained by FORM (Tsakiris, 2010; Blanke et al., 2015).

### Hypothesis three: the effect of offset on embodiment

We hypothesized that OFFSET would not influence embodiment measures in this setup. Although we did find an effect of OFFSET on *embodiment–location*, the subscales of *embodiment–ownership*, and *embodiment–agency* were unaffected by differences in OFFSET (0 vs. 30 cm). This suggests OFFSET is not important for *embodiment–ownership* or *embodiment–agency*, at least not when the artificial hand is viewed near the trunk and the offset is within 45 cm (Zopf et al., 2010; Preston, 2013).

### Hypothesis four: the interaction effect of touch and offset on embodiment

Contrary to our expectations, the effect of TOUCH on embodiment did not increase when we introduced a spatial offset between participants viewed and actual hands. This contrasted with earlier findings from our lab, which suggested that increasing lateral distance might increase the influence of TOUCH in the RHI (Zopf et al., 2010), although significant interactions between TOUCH and OFFSET were also not reported in that study. Our previous study employed an even larger distance and compared a 45 cm offset with a 15 cm offset. It is possible that the effect of TOUCH is greater for offsets beyond 30 cm. Taken together, these findings indicate that TOUCH has a direct effect on embodiment independent of the FORM or OFFSET of the depicted hand feedback.

### Hypothesis five: the effect of touch occurrence on sense of agency

As expected, contrasts between touch and no touch conditions showed that touch occurrence contributed to significant higher scores for all agency measures. Although, participants' hands remained still, tactile sensations appear to facilitate the perception of action, and a sense of control over one's actions in the experiment. However, there was no effect of touch synchrony, suggesting that when a touch does occur, agency measures are insensitive to temporal delays.

### The effect of TOUCH, FORM, and OFFSET on presence

We had no strong predictions for the effect of different cues on presence and this part of our study was explorative. We found a significant impact of FORM and TOUCH, as well as both touch occurrence and touch synchrony on *presence*. Furthermore, we found non-significant trends for an OFFSET effect (*p* = 0.098) as well as for an interaction between FORM and OFFSET (*p* = 0.077). This suggests that the experience of *presence* in our VR setup is significantly modulated by the cues that also influence embodiment. This implies in turn, that these cues influence the experience of being situated in a virtual environment, in addition to direct experience of one's own body.

### Complex pattern of influence of cues on components of self-representation

Overall, univariate ANOVAs for each rating measure revealed that FORM, TOUCH, and OFFSET influenced different components of self-representation. FORM had a significant impact on all *embodiment* subscales, *effortlessness*, and *presence*. TOUCH had a significant influence on all *embodiment* subscales, *involuntariness* and *presence*. OFFSET had a significant impact on the *embodiment–location* subscale only. Furthermore, touch occurrence had a significant impact on all rating scales, whereas touch synchrony did not significantly impact any of the *agency* scales

In line with previous work, we found no effect of OFFSET on *embodiment–ownership* (Zopf et al., 2010; Kilteni et al., 2012; Preston, 2013). However, we did find that the experience of location for one's own body was significantly affected by OFFSET when directly comparing a no spatial offset with a spatial offset condition. So in contrast to the other components of self-experience, *embodiment–location* was sensitive to a spatial difference between visual- and proprioceptive location information in the virtual hand illusion. This supports the idea that *embodiment–ownership* and *embodiment–location* correspond to different self-components with different mechanisms (Serino et al., 2013).

The pattern of results for *presence* ratings suggests that *presence* tends to be influenced by similar cues as *embodiment-location* ratings (although for OFFSET there were trends for significance only). The current findings suggest that this shift toward prioritizing virtual environment cues over real environment cues is facilitated when there is a visual hand form, multisensory touch signals, and no conflict between the perceived spatial location of an individual's virtual body and the actual location of their real body.

For agency measures, we found an effect of touch occurrence. However, these agency measures were not modulated by touch synchrony. This accords with the previous finding that visual-tactile synchrony affects different components of self-representation such as ownership, location and agency differently (Longo et al., 2008b; Kalckert and Ehrsson, 2012). However, movement synchrony has previously been shown to affect agency ratings (Kalckert and Ehrsson, 2012). Agency seems sensitive to movement synchrony but not to touch synchrony when the hand is passive. Additionally, agency scores were not affected by OFFSET. Agency therefore seems robust to both temporal and spatial multisensory discrepancies, whereas the other self-representation components were not. This is in line with research showing agency can be experienced for spatially and temporally distant events (Faro et al., 2013). However, agency was not immune to all sensory cues. FORM significantly increased *embodiment-agency* as well as *effortlessness* ratings, suggesting that participants were more likely to experience agency for a target that was visually congruent with their own body. Thus overall, visual information and FORM congruency had a significant influence on all measures of self-representation.

Not all agency rating scales were affected by FORM. We found no significant effect on *involuntariness*. In this study we used the SOARS, which conceptualizes sense of agency as comprising two primary dimensions: *involuntariness* and *effortlessness*; and also the *embodiment-agency* subscale, which conceptualizes sense of agency as a subcomponent of embodiment. In earlier work, Polito et al. (2013) showed that *involuntariness* and *effortlessness* are quite distinct conceptual subcomponents of the subjective sense of agency. It may be that *effortlessness* (and also *embodiment–agency*) tap processes related to monitoring of sensory signals, including visual cues; whereas *involuntariness* taps more attributional judgments about agentive experience: for example, tracking whether a movement actually occurred (there were no actual self-generated movements in this task).

This componential view of agency is consistent with research indicating that sense of agency is a multidimensional construct that fluctuates in response to a range of sensory and cognitive signals over time and across domains (Synofzik et al., 2008; Gallagher, 2012; Polito et al., 2014). The current results suggest that body-congruent visual cues may influence the immediate, felt experience of agency (represented by higher *effortlessness* scores), whereas the sensation of touch may influence attributional judgments of agency (represented by higher *involuntariness* scores).

To summarize, these findings highlight similarities and differences between *ownership, location, presence* and the three agency aspects *embodiment–agency, effortlessness*, and *involuntariness*. Based on the findings here, there is some overlap but also important differences between the influence of different cues on these components.

### No single cue strictly constrains self-representation in the VHI

The common link between the three cues we manipulated is that they all involve comparing a condition where visual information is in harmony with other bodily information, to a condition where a discrepancy is introduced: whether a viewed body form matches an actual body form; whether a viewed touch corresponds to a felt touch, and whether a viewed hand position matches the proprioceptively felt hand position. In all three cases, a better match generally signals that the visual information is more plausible and therefore more likely to be related to one's own body.

Previous accounts of body ownership and self-consciousness proposed that specific cues can operate as strict hierarchical constraints on the processing of subsequent cues (e.g., Tsakiris, 2010; Blanke et al., 2015). For example, one influential model of body-ownership posits a hierarchical sequence of matching stages in which successful matching at one stage permits matching at the next stage, and unsuccessful matching gates or constrains further processing stages (Tsakiris, 2010). According to this account, in the first stage, current visual information about form is matched with a stored model of the way the body typically looks to eliminate gross mismatches. Only if matching is successful in the first stage is a second stage of more fine-grained comparisons performed between visual and proprioceptive information about bodily posture and anatomical position. Finally, only if a postural match is confirmed in the second stage, does a third stage of comparisons commence in which the temporal synchrony between viewed and felt touch is analyzed. According to this model, because matching at each stage is hypothesized to occur in a strict hierarchy, a form mismatch, for example, will restrict or gate the sense of body ownership even if other cue comparisons such as visual-tactile synchrony suggest congruency. This model therefore predicts specific interactions between the different cues involved in the various comparison stages.

We found no evidence for strict hierarchical interactions. Instead, we primarily observed main effects for different cues on self-representation. This implies that whereas each of the cues is important for self-representation, none hierarchically constrains or limits the influence of any of the other cues. Congruent information from all types of cue can, to some extent, independently and non-hierarchically influence self-representation. This finding indicates a flexible self-representation system that can readily adapt to different combinations of multisensory cues.

### Implications for VR methods

Overall, this study demonstrated that consumer-grade VR equipment can be used in the lab to investigate cues that influence self-representation. Studying self-representation in VR allows for a high level of experimental control, continuity, and accurate repeatability of stimulus presentation. We have successfully set up a VR laboratory environment using less than AU$1000 in VR hardware and software (not including PC equipment), that allowed us to manipulate visual, tactile, and proprioceptive cues. Equipment and software is readily available, with a number of consumer VR HMD vendors entering the market in 2016.

We successfully demonstrated a calibration procedure for appropriately registering the virtual environment as viewed by each participant so that it aligned with the real environment. We achieved this by measuring participant inter-pupillary distance, determining the ratio between units of measurement in the virtual environment and real world measurements, and by appropriately sizing virtual objects to achieve a good match. By correctly locating the HMD in real space, we can, with a single keypress, move the virtual viewpoint to the corresponding position. Good calibration is important for avoiding unwanted or unmeasured experimental influences. Following calibration, the built-in head position tracking of the VR system ensures that the participant's virtual viewpoint is thereafter constantly aligned to their real head and view position. This procedure demonstrates the simplicity with which consumer VR systems can be used for research where a requirement is close calibration between real and virtual environment features.

Our findings also have implications for human-computer interface design and a variety of consumer VR applications. VR software designers aim to create virtual worlds, games and experiences that distinguish their software from conventional 2D software. This means maximizing user experiences of presence, embodiment, and agency over virtual avatars. Understanding the relationships between specific sensory cues and users' subjective self-representations can inform this intention, giving developers more detailed information on the features and controls important for achieving good design. There are five findings from this study that may inform VR applications. First, that both visual form congruency and touch synchrony are generally important for compelling self-representation in VR. Second, relative to those cues, a spatial discrepancy between the proprioceptively felt real hand location and the visually apparent virtual hand location is not a sensitive influence on most elements of self-representation. Third, agency and presence seem to depend on the same multisensory cues (FORM, TOUCH, and OFFSET) that have been identified as important in the embodiment literature. Fourth, touch stimuli can be used in different ways: synchronous touch influences feelings of embodiment and presence, whereas the simple occurrence of touch may be sufficient to influence a sense of agency. Fifth, cues differ in their relevance for different components of self-experience in VR. So, depending on what self-experience is important for a specific VR implementation or product (e.g., ownership vs. agency), the designer may focus on different cues. Furthermore, these results can inform the design of VR software for therapeutical settings, where modulating the intensity of self-representation with different cues (e.g., employing graduated exposure treatments in anxiety disorders) could be important.

### Limitations

An important innovation of our study was that we investigated the influence of a set of cues on a set of components thought to be important for self-representation. To do this we employed rating scales. Rating scales require participants to make explicit judgment responses and these may be subject to responses biases. For example, participants may have responded to different rating scales in a similar manner or responded to the repetition of the questionnaires similarly. We tried to provide a safe-guard for repetitive response patterns by randomizing the rating scales. That we found different patterns for different rating scales suggests that we did tap into differences in self-representation that were not simply due to the way participants tended to respond to these items. In future research, converging evidence from implicit measures will be useful to further investigate the mechanisms that support the representation of one's own body and actions.

For presence we only employed a small set of ratings (Sanchez-Vives and Slater, 2005). In future studies a full presence rating scale measure could be used (e.g., Lessiter et al., 2001; but see Slater et al., 2009b, for a critique of questionnaires for measuring presence, and suggested alternatives such as physiological measures).

In this study we manipulated a combination of visual, tactile and proprioceptive cues while the body was static. However, in many real-world scenarios as well as VR-applications the body is moving. Additional cues related to initiating a movement and processing movement feedback are likely crucial for self-representation, particularly for agency. To further study these cues and interactions with FORM, TOUCH, and OFFSET on several aspects of self-representation, an active Virtual Hand Illusion paradigm could be implemented.

Lastly, in our experimental design there is room for improvement in achieving synchrony between visual and tactile feedback relating to the experience of a touch. Since the system delay from the onset of visual feedback to onset of the tactor vibration is so steady, hardware based delays could be overcome by hard-coding a countering delay for the visual feedback, such that delay between the two is extinguished.

## Conclusion

Our findings shed light on the multivariate influence of visual form congruency (whether the virtual hand appears similar in form to the participant's real hand), touch synchrony (whether virtual visual feedback about touch is temporally synchronized with physically experienced sensations of touch) and hand position alignment (whether or not visual and proprioceptive feedback about hand position are in agreement) on participants' experiences of embodiment, presence and sense of agency. We provided evidence that each type of cue can independently influence self-representation, but that none of these cues strictly constrains or gates the influence of the others. We also demonstrated that consumer-grade VR equipment can be used successfully in the cognitive and brain sciences to investigate self-representation.

## Author contributions

All authors together designed the experiments; SP setup the VR-equipment and programmed the experiments; SP and VP collected the data; RZ and VP analyzed the data; SP, VP, and RZ wrote the paper with input from DK and MW.

### Conflict of interest statement

The authors declare that the research was conducted in the absence of any commercial or financial relationships that could be construed as a potential conflict of interest.

## Footnotes

^1^http://www.oculus.com/en-us/dk2/.

^2^http://unity3d.com/. Unity 3D allows a number of options for writing supporting programming code. For these experiments we used the C# programming language, which is widely used within the Unity3D user community.

^3^The hand model was obtained from the Leap Motion Unity core assets package, available at: http://developer.leapmotion.com/unity.

^4^Note: the configuration utility was available for the Oculus DK2 and in the developmental Oculus runtime version 0.6.0.1 as used in our experiments. However, in the commercial release of the Oculus Rift and runtime, this configuration utility is no longer available. IPD adjustments for the commercial release are now made with a slider control on the underside of the commercial release Oculus Rift HMD.

## References

[B1] Apps M. A. Tsakiris M. (2014). The free-energy self: a predictive coding account of self-recognition. Neurosci. Biobehav. Rev. 41, 85–97. 10.1016/j.neubiorev.2013.01.029 23416066 PMC3848896

[B2] Armel K. C. Ramachandran V. S. (2003). Projecting sensations to external objects: evidence from skin conductance response. Proc. R. Soc. Lond. B Biol. Sci. 270, 1499–1506. 10.1098/rspb.2003.2364 12965016 PMC1691405

[B3] Aspell J. E. Lenggenhager B. Blanke O. (2009). Keeping in touch with one's self: multisensory mechanisms of self-consciousness. PLoS ONE 4:e6488. 10.1371/journal.pone.0006488 19654862 PMC2715165

[B4] Barfield W. Hendrix C. (1995). The effect of update rate on the sense of presence within virtual environments. Virtual Real. 1, 3–15. 10.1007/BF02009709

[B5] Barfield W. Hendrix C. Bystrom K.-E. (1999). Effects of stereopsis and head tracking on performance using desktop virtual environment displays. Presence 8, 237–240. 10.1162/105474699566198

[B6] Blakemore S. J. Wolpert D. Frith C. (2000). Why can't you tickle yourself? Neuroreport 11, R11–R16. 10.1097/00001756-200008030-00002 10943682

[B7] Blanke O. (2012). Multisensory brain mechanisms of bodily self-consciousness. Nat. Rev. Neurosci. 13, 556–571. 10.1038/nrn3292 22805909

[B8] Blanke O. Slater M. Serino A. (2015). Behavioral, neural, and computational principles of bodily self-consciousness. Neuron 88, 145–166. 10.1016/j.neuron.2015.09.029 26447578

[B9] Botvinick M. Cohen J. (1998). Rubber hands ‘feel’ touch that eyes see. Nature 391, 756. 10.1038/35784 9486643

[B10] Bowers K. S. (1981). Do the Stanford scales tap the 'classic suggestion effect'? Int. J. Clin. Exp. Hypn. 29, 42. 10.1080/00207148108409142 7275362

[B11] Ehrsson H. H. (2012). The concept of body ownership and its relation to multisensory integration, in The New Handbook of Multisensory Processing, ed Stein B. E. (Cambridge, MA: MIT Press). 775–792.

[B12] Faro D. McGill A. L. Hastie R. (2013). The influence of perceived causation on judgments of time: an integrative review and implications for decision-making. Front. Psychol. 4:217. 10.3389/fpsyg.2013.00217 23717286 PMC3653058

[B13] Field A. (2009). Discovering Statistics with SPSS, 3rd Edn. London: Sage.

[B14] Frith C. D. Done D. J. (1989). Experiences of alien control in schizophrenia reflect a disorder in the central monitoring of action. Psychol. Med. 19, 359–363. 10.1017/S003329170001240X 2762440

[B15] Gallagher S. (2012). Multiple aspects in the sense of agency. New Ideas Psychol. 30, 15–31. 10.1016/j.newideapsych.2010.03.003

[B16] Haggard P. Clark S. Kalogeras J. (2002). Voluntary action and conscious awareness. Nat. Neurosci. 5, 382–385. 10.1038/nn827 11896397

[B17] Hendrix C. Barfield W. (1995). Presence in virtual environments as a function of visual and auditory cues, in Paper Presented at the Proceedings of the 1995 IEEE Annual Virtual Reality International Symposium, Triangle Park, NC.

[B18] Ijsselsteijn W. A. Freeman J. de Ridder H. (2001). Presence: where are we? Cyberpsychol. Behav. 4, 179–182. 10.1089/109493101300117875 11710245

[B19] Kalckert A. Ehrsson H. H. (2012). Moving a rubber hand that feels like your own: a dissociation of ownership and agency. Front. Hum. Neurosci. 6:40. 10.3389/fnhum.2012.00040 22435056 PMC3303087

[B20] Khanna P. Yu I. Mortensen J. Slater M. (2006). Presence in response to dynamic visual realism: a preliminary report of an experiment study, in Paper Presented at the 13th ACM Symposium Virtual Reality Software and Technology, VRST'06, Limassol.

[B21] Kihlstrom J. F. (2008). The domain of hypnosis, revisited, in The Oxford Handbook of Hypnosis: Theory, Research and Practice, eds Nash M. R. Barnier A. J. (Oxford: Oxford University Press), 21–52.

[B22] Kilteni K. Maselli A. Koerding K. P. Slater M. (2015). Over my fake body: body ownership illusions for studying the multisensory basis of own-body perception. Front. Hum. Neurosci. 9:141. 10.3389/fnhum.2015.00141 25852524 PMC4371812

[B23] Kilteni K. Normand J. M. Sanchez-Vives M. V. Slater M. (2012). Extending body space in immersive virtual reality: a very long arm illusion. PLoS ONE 7:e40867. 10.1371/journal.pone.0040867 22829891 PMC3400672

[B24] Lessiter J. Freeman J. Keogh E. Davidoff J. (2001). A cross-media presence questionnaire: the ITC-sense of presence inventory. Presence 10, 282–297. 10.1162/105474601300343612

[B25] Longo M. R. Cardozo S. Haggard P. (2008a). Visual enhancement of touch and the bodily self. Conscious. Cogn. 17, 1181–1191. 10.1016/j.concog.2008.01.001 18294867

[B26] Longo M. R. Schüür F. Kammers M. P. Tsakiris M. Haggard P. (2008b). What is embodiment? A psychometric approach. Cognition 107, 978–998. 10.1016/j.cognition.2007.12.004 18262508

[B27] Ma K. Hommel B. (2015). Body-ownership for actively operated non-corporeal objects. Conscious. Cogn. 36, 75–86. 10.1016/j.concog.2015.06.003 26094223

[B28] Moore J. W. Fletcher P. C. (2012). Sense of agency in health and disease: a review of cue integration approaches. Conscious. Cogn. 21, 59–68. 10.1016/j.concog.2011.08.010 21920777 PMC3315009

[B29] Morgan H. L. (2015). Sense of agency and sense of ownership: arguing against a dissociation and for a critical role for multisensory binding. Psychol. Conscious. 2, 222–236. 10.1037/cns0000069

[B30] Oculus V. R. (2016). Oculus Best Practices. Available online at: http://static.oculus.com/documentation/pdfs/intro-vr/latest/bp.pdf

[B31] Polito V. Barnier A. J. Woody E. Z. (2013). Developing the Sense of Agency Rating Scale (SOARS): an empirical measure of agency disruption in hypnosis. Conscious. Cogn. 22, 684–696. 10.1016/j.concog.2013.04.003 23685619

[B32] Polito V. Barnier A. J. Woody E. Z. Connors M. H. (2014). Measuring agency change across the domain of hypnosis. Psychol. Conscious. 1, 3–19. 10.1037/cns0000010

[B33] Polito V. Waters F. A. McIlwain D. (2015). Sense of agency: theory, methods, and application. Psychol. Conscious. 2, 207–209. 10.1037/cns0000073

[B34] Preston C. (2013). The role of distance from the body and distance from the real hand in ownership and disownership during the rubber hand illusion. Acta Psychol. (Amst). 142, 177–183. 10.1016/j.actpsy.2012.12.005 23333877

[B35] Rohde M. Di Luca M. Ernst M. O. (2011). The rubber hand illusion: feeling of ownership and proprioceptive drift do not go hand in hand. PLoS ONE 6:e21659. 10.1371/journal.pone.0021659 21738756 PMC3125296

[B36] Sanchez-Vives M. V. Slater M. (2005). From presence to consciousness through virtual reality. Nat. Rev. Neurosci. 6, 332–339. 10.1038/nrn1651 15803164

[B37] Serino A. Alsmith A. Costantini M. Mandrigin A. Tajadura-Jimenez A. Lopez C. (2013). Bodily ownership and self-location: components of bodily self-consciousness. Conscious. Cogn. 22, 1239–1252. 10.1016/j.concog.2013.08.013 24025475

[B38] Shimada S. Fukuda K. Hiraki K. (2009). Rubber hand illusion under delayed visual feedback. PLoS ONE 4:e6185. 10.1371/journal.pone.0006185 19587780 PMC2702687

[B39] Slater M. Khanna P. Mortensen J. Yu I. (2009a). Visual realism enhances realistic response in an immersive virtual environment. IEEE Comput. Graph. Appl. 29, 76–84. 10.1109/MCG.2009.55 19642617

[B40] Slater M. Lotto B. Arnold M. M. Sanchez-Vives M. V. (2009b). How we experience immersive virtual environments: the concept of presence and its measurement. Anuario Psicol. 40, 193–210.

[B41] Slater M. Perez-Marcos D. Ehrsson H. H. Sanchez-Vives M. V. (2008). Towards a digital body: the virtual arm illusion. Front. Hum. Neurosci. 2:6. 10.3389/neuro.09.006.2008 18958207 PMC2572198

[B42] Synofzik M. Vosgerau G. Newen A. (2008). I move, therefore I am: a new theoretical framework to investigate agency and ownership. Conscious. Cogn. 17, 411–424. 10.1016/j.concog.2008.03.008 18411059

[B43] Tsakiris M. (2010). My body in the brain: a neurocognitive model of body-ownership. Neuropsychologia 48, 703–712. 10.1016/j.neuropsychologia.2009.09.034 19819247

[B44] Tsakiris M. Carpenter L. James D. Fotopoulou A. (2010). Hands only illusion: multisensory integration elicits sense of ownership for body parts but not for non-corporeal objects. Exp. Brain Res. 204, 343–352. 10.1007/s00221-009-2039-3 19820918

[B45] Tsakiris M. Haggard P. (2005). The rubber hand illusion revisited: visuotactile integration and self-attribution. J. Exp. Psychol. 31, 80–91. 10.1037/0096-1523.31.1.80 15709864

[B46] Tsakiris M. Prabhu G. Haggard P. (2006). Having a body versus moving your body: how agency structures body-ownership. Conscious. Cogn. 15, 423–432. 10.1016/j.concog.2005.09.004 16343947

[B47] Wegner D. M. (2004). Précis of the illusion of conscious will. Behav. Brain Sci. 27, 649–659. 10.1017/S0140525X04000159 15895616

[B48] Wegner D. M. Wheatley T. (1999). Apparent mental causation. Sources of the experience of will. Am. Psychol. 54, 480–492. 10.1037/0003-066X.54.7.480 10424155

[B49] Yu I. Mortensen J. Khanna P. Spanlang B. Slater M. (2012). Visual realism enhances realistic response in an immersive virtual environment - Part 2. IEEE Comput. Graph. Appl. 32, 36–45. 10.1109/MCG.2012.121 24807308

[B50] Zhang J. Chen W. Li H. Hommel B. Yuan T.-F. (2014). Disentangling the sense of agency and the sense of ownership in the virtual hand illusion paradigm. PeerJ Preprints. 2:e673v1. 10.7287/peerj.preprints.673v1

[B51] Zopf R. Savage G. Williams M. A. (2010). Crossmodal congruency measures of lateral distance effects on the rubber hand illusion. Neuropsychologia 48, 713–725. 10.1016/j.neuropsychologia.2009.10.028 19913040

[B52] Zopf R. Savage G. Williams M. A. (2013). The crossmodal congruency task as a means to obtain an objective behavioral measure in the rubber hand illusion paradigm. J. Vis. Exp. e50530 10.3791/50530 PMC3846377 23912051

[B53] Zopf R. Truong S. Finkbeiner M. Friedman J. Williams M. A. (2011). Viewing and feeling touch modulates hand position for reaching. Neuropsychologia 49, 1287–1293. 10.1016/j.neuropsychologia.2011.02.012 21320514 PMC3086579

