# Supplementary material for: Non-hierarchical Influence of Visual Form, Touch, and Position Cues on Embodiment, Agency, and Presence in Virtual Reality
Source: Front Psychol. 2016 Oct 25;7:1649. doi: 10.3389/fpsyg.2016.01649 (PMC5078469; doi:10.3389/fpsyg.2016.01649)
Supplement: Supplementary file 1 [file DataSheet1.docx]

# Appendix A: Oculus Rift DK2 specifications

| Release Date | 24 July 2014 | [http://www.oculusvr.com](http://www.oculusvr.com/) |
| --- | --- | --- |
| Cost | USD350 + delivery | [http://www.oculusvr.com](http://www.oculusvr.com/) |
| Weight | 440 grams | <http://www.theverge.com/products/oculus-rift-dk2/7646> |
| Nominal field-of-view | 100 degrees, diagonal | http[://vrwiki.wikispaces.com/Oculus+Rift+Development+Kit+2](https://vrwiki.wikispaces.com/Oculus+Rift+Development+Kit+2) |
| Screen technology | Low persistence Super AMOLED (same as Samsung Galaxy Note 3) | <http://www.roadtovr.com/samsung-galaxy-note-3-touchscreen-inside-oculus-rift-dk2/> |
| Screen size | 5.7" | <http://www.ifixit.com/Device/Oculus_Rift_Development_Kit_2> |
| Screen resolution | 1080p (1920 x 1080) | <http://www.ifixit.com/Device/Oculus_Rift_Development_Kit_2> |
| Screen resolution per eye | 960 x 1080 | <http://www.ifixit.com/Device/Oculus_Rift_Development_Kit_2> |
| Pixel layout | Pentile | <http://en.wikipedia.org/wiki/PenTile_matrix_family> |
| Screen refresh rate | 75 Hz | [http://www.oculusvr.com](http://www.oculusvr.com/) |
| Tracking | 6 degrees of freedom | [http://www.oculusvr.com](http://www.oculusvr.com/) |
| Rotational tracking technology | Gyroscope/ accelerometer/ magnetometer | <http://riftinfo.com/oculus-rift-specs-dk1-vs-dk2-comparison> |
| Rotational tracking sampling rate | 1,000 Hertz | <http://riftinfo.com/oculus-rift-specs-dk1-vs-dk2-comparison> |
| Positional tracking | Near IR CMOS sensor camera | <http://riftinfo.com/oculus-rift-specs-dk1-vs-dk2-comparison> |
| Tracking camera resolution | 752 x 480 | <https://vrwiki.wikispaces.com/Oculus+Rift+Development+Kit+2> |
| No. of IR LEDs on headset (for positional tracking) | 40 | <https://vrwiki.wikispaces.com/Oculus+Rift+Development+Kit+2> |
| Positional tracking sampling rate | 60 Hz | <http://riftinfo.com/oculus-rift-specs-dk1-vs-dk2-comparison> |
| Positional tracking range of movement | 0.5 metres to 2.5 metres | <https://vrwiki.wikispaces.com/Oculus+Rift+Development+Kit+2> |
| Positional tracking camera field-of-view | 72 degrees wide, 52 degrees high | <https://vrwiki.wikispaces.com/Oculus+Rift+Development+Kit+2> |
| Positional tracking precision | 0.05mm at 1.5 metres | <https://vrwiki.wikispaces.com/Oculus+Rift+Development+Kit+2> |
| Optics | Aspherical polycarbonate lenses | <https://vrwiki.wikispaces.com/Oculus+Rift+Development+Kit+2> |
| Interaxial distance btw. lenses | Fixed at 63.5 mm | <http://developer.oculus.com/documentation/intro-vr/latest/concepts/bp_app_imaging/> |
| Effective screen distance from eyes via optics | 1.3 metres | <http://developer.oculus.com/documentation/intro-vr/latest/concepts/bp_app_imaging/> |

# Appendix B: Other hardware/software specifications

| Central Processing Unit | Intel i5-4590 @ 3.3GHz | <http://ark.intel.com/products/80815/Intel-Core-i5-4590-Processor-6M-Cache-up-to-3_70-GHz> |
| --- | --- | --- |
| RAM | 16 Gigabytes |  |
| Graphical Processing Unit | Nvidia GeForce GTX 970 | <http://www.geforce.com/hardware/desktop-gpus/geforce-gtx-970> |
| Sound card (to drive tactor) | Creative Sound Blaster X-fi Titanium HD | <http://www.soundblaster.com/products/Sound-Blaster-X-Fi-Titanium-HD.aspx> |
| Tactor setup | Dancer Design tactor, driven by 2-channel mini tactamp | <http://www.dancerdesign.co.uk/> |
| Operating System | Windows 8.1 Enterprise 64-bit | <http://www.microsoft.com/> |
